# Identification of diacetonamine from soybean curd residue as a sporulation-inducing factor toward *Bacillus* spp.

**DOI:** 10.1186/s13568-017-0395-0

**Published:** 2017-05-23

**Authors:** Aki Ikeda, Dongyeop Kim, Yasuyuki Hashidoko

**Affiliations:** 1 Research Faculty of Agriculture, Hokkaido University, Kita 9 Nishi 9, Kita-Ku, Sapporo, 060-8589 Japan; 2 School of Dental Medicine, University of Pennsylvania, 240 South 40th Street, Philadelphia, PA 19104 USA

**Keywords:** Sporulation-inducing factor, Soybean curd residue, *Bacillus amyloliquefaciens*, *Bacillus megaterium*, Diacetonamine, Diacetone acrylamide

## Abstract

Under bioassay-guided investigation, a sporulation-inducing factor (SIF) toward *Bacillus* spp. was searched for in methanol (MeOH) extracts of soybean curd residues, and diacetonamine (**1**) was identified as the active compound. SIF was first isolated as a monoacetylated derivative (**2**, 4.1 mg from 655 g soybean curd residues), and its chemical structure was elucidated by field desorption mass spectrometry, electron ionization mass spectrometry, and nuclear magnetic resonance (NMR) analyses. After 48-h incubation, 40 µM diacetonamine hydrochloride (**1b**) exhibited sporulation-inducing activity with 35% sporulation frequency toward a *Bacillus amyloliquefaciens* wild-type strain (AHU 2170), whereas 40 µM diacetone acrylamide (**3**) showed 99% sporulation induction, which was much higher than that of **1b**. Although *Bacillus megaterium* NBRC 15308 was sporulated by the treatment with 400 µM **1b** with 36 and 70% sporulation frequency after 72- and 96-h incubation respectively, **3** at the same concentration showed only 2% sporulation after 72-h incubation. Hence, diacetonamine (**1**) was characterized as a genuine SIF from soybean curd residues, but it was uncertain whether **1** is a natural product or an artifact. Spores of *B. amyloliquefaciens* induced by **1b** survived after treatment with heating at 95 °C for 10 min, also suggesting that **1** is genuine SIF in soybean curd residue. As sporulation induction is likely linked to activation of antibiotic production in some spore-forming *Firmicutes* bacteria, compound **1** would be a possible chemical tool to develop an effective fermentation technology in *Bacillus* species.

**Electronic supplementary material:**

The online version of this article (doi:10.1186/s13568-017-0395-0) contains supplementary material, which is available to authorized users.

## Introduction

Few genera of the phylum *Firmicutes*, such as *Bacillus*, *Paenibacillus*, and *Clostridium*, form endospores under nutrient-starvation conditions, an event known as sporulation (Higgins and Dworkin 2012). Bacterial spores are highly tolerant to heat or drought, and even survive after autoclaving (e.g. 120 °C for 15 min). Many functional genes for sporulation in *Bacillus* spp. have been investigated (Eijlander et al. 2014). These spore-forming Gram-positive bacteria show not only morphological but also metabolic differentiation. In metabolic differentiation, active production of BT-toxin in *B. thuringiensis* (de Maagd et al. 2001; Deng et al. 2014) and some lipopeptide antibiotics in *B. subtilis* antibiotic production (Stein 2005), for example, are highly linked to their sporulation events. Not only severe starvation stresses but also some other physical and physiological stimulants can lead to sporulation induction (de Vries et al. 2004; Rashad et al. 2013), and this mechanism appears similar to biofilm formation for Gram-negative bacteria as a starvation-tolerant state, of which morpho-differentiation and metabolic changes are regulated by auto-regulatory chemical signals known as quormones or autoinducers (Davies et al. 1998; Duanis-Assaf et al. 2015). Soybean curd residue, also known as soybean pulp or okara, is an excellent culture medium ingredient for antibiotic production in several antibiotic-producing microorganisms (Hayashi et al. 1989; O’Toole 1999; Mizumoto et al. 2006). Particularly, *Bacillus subtilis* produces a large amount of iturins, cyclic lipopeptide antibiotics, in soybean curd residue medium (Ohno et al. 1995, 1996). Conversely, numerous reports also suggested a tight link between active sporulation induction and antibiotic production among *Bacillus* species cultured in soybean curd residues (Stein 2005).

**Fig. 1:**
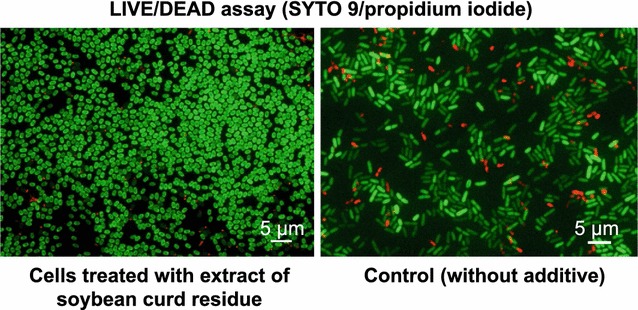
Sporulation induction on vegetative cells of *B. amyloliquefaciens* AHU 2170 by an extract from soybean curd residue. An active fraction obtained by preliminary cation-exchange chromatography induced pellicle along with sporulation induction at a concentration equivalent to 1 g fresh weight of soybean curd residues per mL MSG medium after 48-h incubation (*left*), while control without any additives did not (*right*). Bacterial cells were stained with SYTO 9/propidium iodide for bacterial LIVE/DEAD assay. Note that shapes of the spore cells stained by SYRO 9 are clearly distinguishable from rod-shaped vegetative cells

Indeed, methanolic extract from soybean curd residue induced sporulation during pellicle formation (Fig. 1). Hence, we screened for sporulation inducing factor (SIF) in soybean curd residues using a wild-type *Bacillus amyloliquefaciens* AHU 2170 originally isolated from the rhizoplane of a home garden-grown Chinese cabbage and stocked in our laboratory (Sugita 2008). Its major antifungal substances were iturin A and fengycin B (Malfanova et al. 2012; Yamamoto et al. 2015), but this strain was less active in producing these lipopeptide antibiotics. When the extracts of soybean curd residue were added to 24-h pre-cultured nutrient-broth medium (corresponding to 1 g wet soybean curd residue per mL medium), antibiotic production in the liquid medium was clearly improved in the culture (Additional file 1: Figure S1). More importantly, sporulation of *B. amyloliquefaciens* was also accelerated, after 48-h exposure to the crude extract (Fig. 1). Given the tight linkage between antibiotic production and sporulation induction based on molecular biology analyses (Eijlander et al. 2014; Deng et al. 2014), we first searched for SIF in the extracts of soybean curd residue using *B. amyloliquefaciens* AHU 2170 and *B. megaterium* NBRC 15308 as the test bacilli.

## Materials and methods

### General

Electron ionization mass spectrometry (EI-MS), field desorption mass spectrometry (FD-MS), and field desorption high-resolution mass spectrometry (FD-HR-MS) were conducted using MS spectrometers JMS-T100GCV and JMS-SX-102 (JEOL, Tokyo, Japan). ^1^H-/^13^C-NMR spectra were recorded using a JEOL JNM-EX270 (JEOL, Tokyo, Japan) at 270/67.5 MHz and a Bruker AMX-500 (Bruker, Billerica, MA, USA) at 500/125 MHz.

**Fig. 2:**
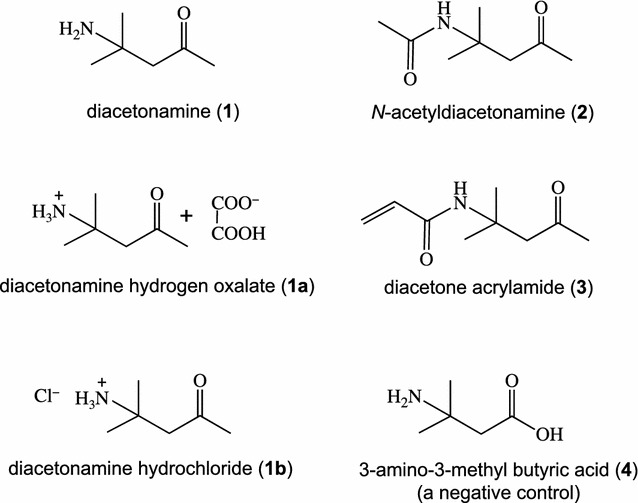
Structures of diacetonamine and its related chemicals

Diacetonamine hydrogen oxalate (**1a**) was purchased from Sigma-Aldrich (St. Louis, MO, USA), and diacetonamine hydrochloride (**1b**) and *N*-acetyldiacetonamine (**2**) were prepared from **1a**. As another chemical derivatizable from **1**, diacetone acrylamide (**3**) was purchased from Wako (Osaka, Japan), while a β-amino acid, 3-amino-3-metylbutyric acid (**4**), used as a negative control was purchased from Combi-Blocks (San Diego, CA, USA). Their chemical structures are shown in Fig. 2.

### Bacterial strains and culture conditions

For the sporulation assay, we used *B. amyloliquefaciens* AHU 2170 isolated from the rhizosphere of Chinese cabbage (*Brassica rapa* var. *pekinensis*) and stocked in our laboratory (Sugita 2008) and *B. megaterium* NBRC 15308 purchased from NITE Biological Resource Center, Kisarazu, Japan. Both strains of bacilli were pre-cultured at 30 °C for 24 h on a nutrient-broth (NB) agar plate before use. The *B. amyloliquefaciens* wild-type strain easily formed spore-based pellicle in NB liquid medium, whereas *B. megaterium* was a rarely sporulating *Bacillus* species (Strnadová et al. 1991; Larsen et al. 2014).

### Bioassay for sporulation

After shaking culture of *B. amyloliquefaciens* or *B. megaterium* in NB medium (40 mL) at 30 °C for 48 h in the dark as reciprocation of 100 rpm, the samples were divided into four portions (10 mL each in 15-mL Falcon tube) and centrifuged at 3500*g* at 4 °C for 15 min. The resulting bacterial cell pellet was re-suspended in 10 mL MSG (minimal salts mixture +0.5% glutamic acid) medium (Freese et al. 1979) containing a test substance equivalent to that originally contained in 10 g soybean curd residue, or maximum concentration at 400 µM. When 400 µM of a chemical solution should be prepared, a 400 mM solution of the compound dissolved in dimethyl sulfoxide (DMSO) was first passed through a sterile Millex^®^-GV membrane filter (0.22 µm polyvinylidene fluoride, Merck Millipore Ltd., Billerica, MA), and a 10 µL portion of the solution was added to 10 mL MSG medium previously autoclaved. The 10-mL cell suspension was linear-shaken in a 30-mL Erlenmeyer flask at 80 rpm at 30 °C for 48 h for *B. amyloliquefaciens*. *B. megaterium* required 72–120 h for sporulation.

A LIVE/DEAD BacLight Bacterial Viability Kit (L13152) (Laflamme et al. 2004) was used to evaluate bacterial sporulation induction. The bacterial cells exposed to a test substance or fraction for the incubation time were collected by brief centrifugation, and the resulting cell pellet was washed with 1 mL saline twice by pipetting and then suspended in 1 mL or 100 µL Milli-Q water. The cell suspension was then mixed with 10 µL of LIVE/DEAD BacLight Bacterial Viability Kit solution (SYTO 9: propidium iodide, 9:1) and incubated for 15 min at room temperature in the dark, and then centrifuged at 10,000*g* for 1 min to remove the supernatant. The resulting cell pellet was re-suspended in 100 or 20 µL Milli-Q water, and a 3-µL portion of the resulting cell suspension was dropped on a glass slide (S1111, Matsunami Glass, Kishiwada, Japan) and pressed tightly with a cover glass (Matsunami Glass). The prepared slide was observed using a fluorescent microscope BioRevo (Keyens, Osaka, Japan) under 1500× magnification with an oil immersion lens.

Spores of *B. amyloliquefaciens* are barrel-like short-rods (physical size of approximately 0.8 µm × 1.2 µm) distinguishable from vegetative cells (typical rods, approximately 0.7 × 2.5 µm). Using a GFP-BP filter (*Ex* 470/40, *Em* 535/50, dichroic mirror 495), live cells and spores were stained as green with SYTO 9, whereas propidium iodide with a TRITC filter (*Ex* 540/25, *Em* 605/70, dichroic mirror 565) stained dead cells red. Competent cells (Yang et al. 2015) were observed as orange-colored rods. The spore cells stained with SYTO 9 were observed as green along the border with the unstained central part. Sporulation frequency (numbers of spore/numbers of spore plus vegetative cells) was calculated.

Vegetative cells of *B. megaterium* are relatively large (1.0 × 15 µm or more), while sporulated cells were more spherical endospores (approximately 0.8 µm × 1.0 µm) than those of *B. amlyloliquefaciens* and easily distinguishable from vegetative cells. Immature spores of *B. megaterium* were stained by SYTO 9, while mature spores were not.

### Heat-tolerance assay for evaluating sporulation frequency

A 100-µL portion of spore suspension diluted 10^1^–10^5^-fold with Milli-Q water in a polymerase chain reaction (PCR) tube was heated at 95 °C for 10 min in a PCR thermocycler. After cooling to room temperature, the suspension was well mixed by pipetting and spread over a NB agar plate using a disposable bacteria spreader. After 24-h incubation at 30 °C in the dark, colonies were counted. As a blank, all bacterial cell suspensions were spread on NB agar plates without heating treatment.

### Extraction of sporulation-inducing factor (SIF) from soybean curd residue

A 10-kg fresh weight soybean curd residue (1.85 kg from Miyoshiya Tofu, Bibai, Japan, 2.45 kg, Mameta, Sapporo, Japan, 3.0 kg, Marukawa Foods, Sapporo, Japan, and 2.7 kg, At the Bran Made of Koyama, Sapporo, Japan) was collected from grocery shops and mixed for extraction with methanol (MeOH). The soybean curd residues were soaked in 10 L of MeOH, and the methanolic extract was collected by decantation and squeezing the remaining residues in a cotton cloth. The squeezed residues were re-extracted with another 6 L of MeOH. The methanolic extracts further collected and combined were filtered in vacuo and concentrated under a low pressure to 500 mL of an aqueous solution. This aqueous solution was partitioned with hexane (500 mL × 3, 0.7 g), ethyl acetate (EtOAc) (500 mL × 3, 2.0 g), and normal butanol (*n*-BuOH) (500 mL × 3, 9.6 g). The resulting aqueous layer was the only fraction active as SIF.

### Column chromatography in Sephadex G-25 and Sephadex LH-20

A half volume of the aqueous layer concentrated to 50 mL (over 25 g) was subjected to gel filtration using a 1.5 L Sephadex G-25 (200 g, GE Healthcare Little Chalfont, UK) column (400 mL per fraction, more than 8 fractions). Sporulation-inducing activities were observed in the first three fractions, from Fr-G-1 to Fr-G-3. The remaining half portions of the extracts were also subjected to gel filtration at the same scale as the Sephadex G-25 column, and all the active fractions obtained were combined (Fr-G-1–3, 21.76 g).

Fraction Fr-G-1–3 was further fractionated by column chromatography using a 450-mL Sephadex LH-20 (Amersham Pharmacia Biotech AB, Uppsala, Sweden) column by eluting with 5% MeOH (300 mL per fraction, total of 12 fractions), and Fr-LH-2 and LH-3 (Fr-LH-2–3, 16.55 g) showed active sporulation induction toward *B. amyloliquefaciens*.

### Cation- and anion-exchange chromatography

For cation-exchange chromatography, Fr-LH-2–3 containing active components were applied to a 600 mL-bed volume of a CM-cellulose (cation exchange carrier, Wako) open column, previously conditioned with 0.2 M aq. HCl followed by washing with Milli-Q water. The active component was eluted with Milli-Q water in the second fraction (600 mL, Fr-CMC-2) obtained as 4.08 g of syrup.

Anion-exchange column chromatography using DEAE-cellulose (Wako, 600 mL-bed volume) was subsequently conducted for Fr-CMC-2. The DEAE-cellulose column, previously conditioned with 0.2 M aq. NaOH solution followed by washing with Milli-Q water, retained the active component in the column, despite elution with Milli-Q water (1200 mL). Therefore, the column was continuously eluted with 600 mL of 0.2 M Na_2_CO_3_ solution. As the concentrated solutes (Fr-DEAE-2, 9.71 g containing excessive Na_2_CO_3_ salts) exhibited sporulation induction, all solutes were subjected to a second gel filtration step in a 400-mL Sephadex G-25 column for elution with Milli-Q water (100 mL for each fraction). The third fraction (Fr-DS-3, 1.02 g) showed sporulation-inducing activity.

### Normal phase silica gel TLC

Fraction Fr-DS-3 was analyzed by normal phase silica gel thin-layer chromatography (TLC) (Merck Kieselgel 60F254 Art. 1.05715, Merck, Kenilworth, NJ, USA) developed in *n*-BuOH:water (H_2_O):acetic acid (AcOH) 4:1:1. The main substance in the fraction gave a dark brown-colored spot at *R*_*f*_ 0.4 by spraying with vanillin-sulfuric acid reagent. The main compound was obtained by preparative TLC (10 × 10 cm) in *n*-BuOH:H_2_O:AcOH 4:1:1 for a small portion of Fr-DS-3. However, the main compound obtained and eluted from silica gel with 50% MeOH showed no sporulation induction. The chemical substance recovered from the plate lower zones of the main band also eluted with 50% MeOH and showed clear sporulation activity.

This fraction exhibited a characteristic unique pinkish color by spraying with *p*-anisaldehyde-sulfuric acid reagent followed by heating, but this compound showed a severe tailing in the acidic solvent. Thus, we replaced AcOH with 25% aq. NH_4_OH solution, and the substance gave a fine spot on normal phase silica gel TLC in *n*-BuOH:25% aq. NH_4_OH:acetone 4:5:2.

### Silica gel column chromatography with *n*-BuOH:25% aq. NH_4_OH:acetone

The active component in Fr-DS-3 (1.00 g) was further purified by silica gel column chromatography (174 g, Wakogel C60, Wako) and eluted with *n*-BuOH: 25% aq. NH_4_OH: acetone (4:5:2). Silica gel column was first conditioned with Milli-Q water followed by the eluting solution, and then Fr-DS-3 was loaded to obtain fractions (25 mL each). Each fraction was immediately concentrated in vacuo. The target substance responsive to *p*-anisaldehyde-sulfuric acid reagent was detected in Fr-15–Fr-22 as a single spot on TLC. All fractions (SIF-Fr-15-22) together (450 mg of a colorless solid) exhibited significant sporulation-inducing activity at a concentration equivalent to 1 g soybean curd residue per mL medium (45 µg SIF-Fr-15-22 per mL).

FD-MS (*m*/*z*, %) of SIF-Fr-15–22 showed several parent ion peaks, such as 407.0 (42), 385.0 (20), 308.1 (21), 215.0 (25), 193.0 (100), and 116.1 (81). We also tested several coloring reagents for the detection of active spots on TLC, including *p*-anisaldehyde-sulfuric acid reagent and ninhydrin. The target compound showed a pale pink color on spraying with *p*-anisaldehyde-sulfuric acid reagent followed by strong heating, but no response to ninhydrin.

### Acetylation of SIF-fraction

Although the active component was highly concentrated in SIF-Fr-15–22, the active fraction contained a relatively high amount of silica gel and contaminants. To remove inactive contaminants, a portion of the dried active fraction was subjected to an acetylation reaction. A portion of SIF-Fr-15–22 (29.5 mg) re-dissolved in 2 mL of acetic anhydride and pyridine (1:1) was incubated at 70 °C for 1 h. One of the main products, **2**, was detected at an *R*_*f*_ 0.15 on silica gel TLC (hexane:EtOAc 1:1) as a purple-colored spot by spraying with *p*-anisaldehyde-sulfuric acid reagent. This product was purified by preparative TLC developed twice in hexane:EtOAc 1:1 to yield 4.0 mg of colorless syrup. EI-MS (*m*/*z*, %): 157 ([M]^+^, 11), 142 ([M–CH_3_]^+^, 9.4), 114 ([M–COCH_2_ + H]^+^, 18), 100 ([M–CH_3_-COCH_2_ + H]^+^, 94), 98 (42), 83 (57), 72 (24), 60 (22), 58 (100), and 43 (85). ^1^H-NMR (270 MHz, δ_H_, in CDCl_3_): 5.8 (1H, br., NH), 2.94 (2H, s, CH_2_), 2.12 (3H, s, CH_3_), 1.90 (3H, s, Ac), and 1.39 (6H, s, 2 × CH_3_). ^13^C-NMR and distorsionless enhancement by polarization transfer (DEPT) (67 MHz, δ_H_, in CDCl_3_): δ_C_ 208.18 (CO), 170.11 (COCH_3_), 52.15 (C), 50.93 (CH_2_), 31.77 (CH_3_), 27.52 (CH_3_ × 2), and 24.36 (COCH_3_).

### Preparation of few diacetonamine derivatives from diacetonamine hydrogen oxalate

Commercially available diacetonamine hydrogen oxalate (**1a**, Sigma-Aldrich) was subjected to an anion exchange reaction. Compound **1a** (1.23 g, 6 mmol) dissolved in 10 mL Milli-Q water was passed through a conditioned DEAE cellulose column (200 mL bed volume). An alkaline fraction eluted first was collected and neutralized using 1 M aq. HCl solution and then concentrated to yield 0.82 g of diacetonamine hydrochloride (**1b**) as a colorless syrup (90% yield).

For the acetylation reaction, **1a** (196.4 mg) dissolved in a 1:1 mixture of Ac_2_O and pyridine (4 mL) was incubated at 70 °C for 1 h. A major product, *N*-acetyldiacetonamine (**2**), was obtained by preparative TLC (134.2 mg, 89% yield) as a pinkish purple-colored spot upon spraying with *p*-anisaldehyde-sulfuric acid spray reagent followed by heating.

### Assay for diacetonamine and its amide derivatives

Diacetonamine hydrochloride (**1b**) was preliminarily tested at concentrations of 4, 40, and 400 µM for sporulation-inducing activities toward *B. amyloliquefaciens* and *B. megaterium*. All the test compounds, including **1b**, *N*-acetyldiacetonamine (**2**), and diacetone acrylamide (**3**), were dissolved in DMSO to be 400 mM, and diluted with MSG medium into final concentrations of 4, 40, and 400 µM. The MSG medium for the sporulation assay always contained 0.1% DMSO.

## Results

### Isolation of SIF and its chemical behaviors

**Scheme 1:**
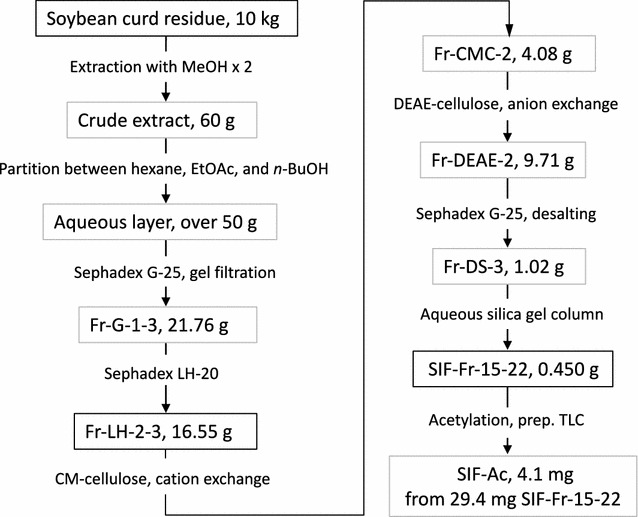
Overall steps for isolation of sporulation-inducing factor from soybean curd residue

The SIF toward *B. amyloliquefaciens* was chased in the extract of soybean curd residue (Scheme 1). Using the sporulation-inducing assay, sporulation-inducing activity was detected in the water-soluble layer, as those equivalent to 10 g soybean curd residue in 10 mL MGS medium. Gel filtration of the water layer (more than 50 g) in Sephadex G-25 column resulted in early elution of the active substance (Fr-G-1–3, total of 21.76 g). Subsequent chromatography for Fr-G-1–3 in Sephadex LH-20 column further fractionated active substance in early fractions (Fr-LH-2–3, 16.55 g).

We attempted cation- and anion-exchange column chromatography for Fr-LH-2–3. By elution with Milli-Q water, the active substance was completely passed through the cation exchange CM-cellulose column, but elution of the active component was clearly retarded from the void fraction (Fr-CMC-2, 4.08 g). The active fraction Fr-CMC-2 was further applied to the anion exchange DEAE-cellulose column. By elution with Milli-Q water, the active substance was captured in the column and eventually eluted with 0.2 M Na_2_CO_3_ solution. After removing Na_2_CO_3_ salt by a second gel filtration step in a Sephadex G-25 column, the active fraction (Fr-DS-3, 1.02 g) gave one major spot on normal phase silica gel TLC (*n*-BuOH:H_2_O:AcOH 4:1:1). However, this acidic and oligomeric sugar-like spot was inactive.

In contrast, a weak purple lower spot on TLC, appearing on spraying with *p*-anisaldehyde-sulfuric acid, was active in the sporulation assay. We further focused on this substance obtained as a fine condensed purple spot on TLC in *n*-BuOH:25% aq. NH_4_OH:acetone 4:5:2. All Fr-DS-3 was subjected to silica gel column chromatography by eluting with the same solvent as used for TLC. The fractions (Fr-15–22) containing the target substance were combined (SIF-Fr-15–22, 450 mg of colorless solid). This fraction yielding the target compound as a single spot on TLC (*n*-BuOH:25% aq. NH_4_OH:acetone 4:5:2) showed clear sporulation induction activity. Approximate calculation by the bioassay suggested that at least 1/10 of the active substance was recovered from the extracts of 10 kg soybean curd residue.

### Identification of diacetonamine as active SIF from soybean curd residue

FD-MS analysis of SIF-Fr-15–22 showed several parent ion peaks, indicating that this active fraction was a mixture. A portion of the active component (29.5 mg) was subjected to acetylation. After this reaction, SFI-Fr-15–22 yielded two major products, and the one detected at *R*_*f*_ 0.15 in hexane:EtOAc 1:1 showed a pinkish color by spraying with *p*-anisaldehyde-sulfuric acid reagent. This target product (4.1 mg of colorless syrup, 30% yield) showed its parent ion at *m*/*z* 157 (115 + COCH_2_), indicating a monoacetylated product derived from compound with a molar mass of 116.1 (chemical formula of C_6_H_13_NO + H^+^, by FD-HR-MS).

^1^H-, ^13^C-NMR, and DEPT spectra of the monoacetylated derivative (**1c**) in chloroform-*d* showed two singlet methyl proton signals at δ_H_ 1.39 magnetically equivalent (CH_3_ × 2, s), singlet methyl proton signal at δ_H_ 1.90 assignable as the acetyl group (–NH–CO–CH_3_), another singlet methyl proton signal at δ_H_ 2.12 assignable as connected with the carbonyl carbon on the skeleton (–CO–CH_3_), and set of methylene proton signals equivalent at δ_H_ 2.94 (s, 2H).

^13^C-NMR and DEPT showed two equivalent methyl carbons at δ_C_ 27.52 (×2), methyl carbon next to the carbonyl group (δ_C_ 31.76), and methylene carbon at δ_C_ 50.93. A quaternary carbon at δ_C_ 52.15 was assigned as connected with an amino group and two methyl groups. Carbon signals at δ_C_ 170.11 (carboxy carbon) and δ_C_ 24.36 (methyl carbon) were assignable to an acetyl group. Thus, one remaining carbon for the chemical formula (C_6_H_13_NO + COCH_2_) was assigned as a carbonyl carbon (δ_C_ 208.18) positioned between the methyl carbon (δ_C_ 31.77) and methylene carbon (δ_C_ 50.93).

The monoacetylated compound, derivatized from the active principle, was thus elucidated as *N*-acetyl-4-amino-4-methyl-2-pentanone (*= N*-acetyldiacetonamine, **2**). Therefore, the compound was identified as diacetonamine (4-amino-4-methyl-2-pentanone, **1**) with a molar mass of 115.1 g/mol. From commercially available diacetonamine hydrogen oxalate (**1a**), *N*-acetyldiacetonamine (**2**) was also prepared (yield 89% from **1a**) and completely matched with **2** prepared from SFI-Fr-15–22 in the ^1^H- and ^13^C-NMR spectra.

### Activity of diacetonamine (1) and diacetonamine amide derivatives

**Fig. 3:**
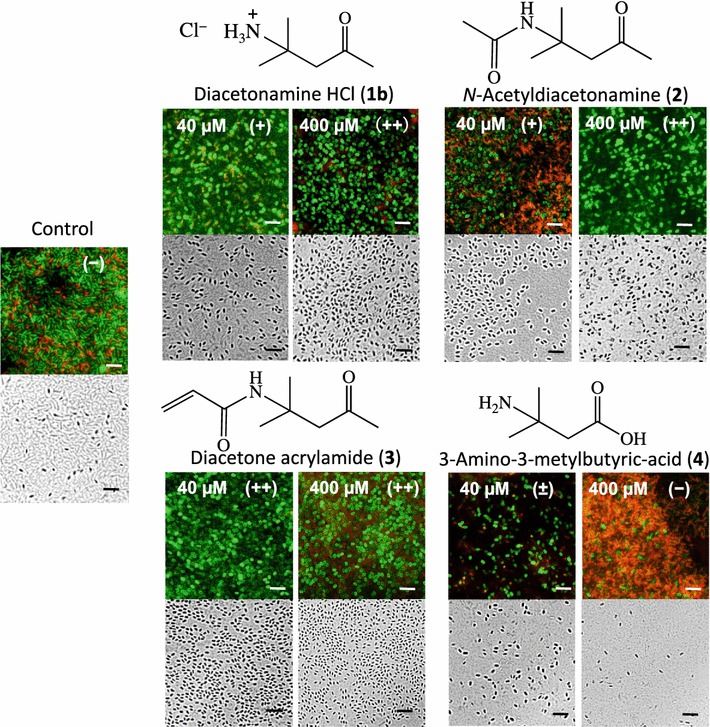
Spore-inducing activity of diacetonamine and its amide derivatives toward *B. amyloliquefaciens* AHU 2170. Sporulation after the treatment with 40 and 400 µM is shown in microscopic photographs. Spores are apparent as short rods stainable as green by SYTO 9. Photo-images are after 48-h incubation upon exposure to the test compounds in MSG medium under a fluorescent microscope. Sporulation frequency was defined as (±), ambiguously positive as less than 10% sporulation from all the vegetative cells; (+), weakly positive as 10–50% sporulation; and (++), positive as >50% sporulation. Scale bar = 5 μm

Using relatively stable diacetonamine hydrochloride (**1b**), the sporulation-inducing activity of **1** was examined. As our isolation process for compound **1** allowed approximate estimation that 1 kg soybean curd residue contains at most 45 mg (approximately 400 µmol) of **1**. Hence, 400 µM was initially set at the concentration of pure **1b** tested for sporulation induction toward the vegetative cells of *B. amyloliquefaciens*. Within 48 h of incubation, compound **1b** at 400 µM showed sporulation inducing activity (51% frequency) nearly equivalent to that of the final active fraction from soybean curd residue, but its 1/10 concentration (40 µM) was less active (35% sporulation frequency) (Fig. 3; Table 1).

**Table 1:** Sporulation frequency of *B. amyloliquefaciens* and *B. megaterium*

Species (conditions)	*B*. *amyloliquefaciens* (%, 48 h)	*B*. *megaterium* (%, 72 h)	*B*. *megaterium* (%, 96 h)
Compound (concentration)			
**1b** (40 µM)	35	–	55
**1b** (400 µM)	51	36	70
**2** (400 µM)	54	−	−
**3** (40 µM)	99	−	85
**3** (400 µM)	69	2	66
**4** (400 µM)	6	−	−
Control	1	0	7

Sporulation frequency (%) is calculated by counting spores and vegetative cells in randomly selected different views. (−) Not tested. More than 300 cells were counted and classified into spores and vegetative cells. For **1b** and **3**, sporulation assays were repeated trice to confirm their reproducible sporulation inducing activities

**Fig. 4:**
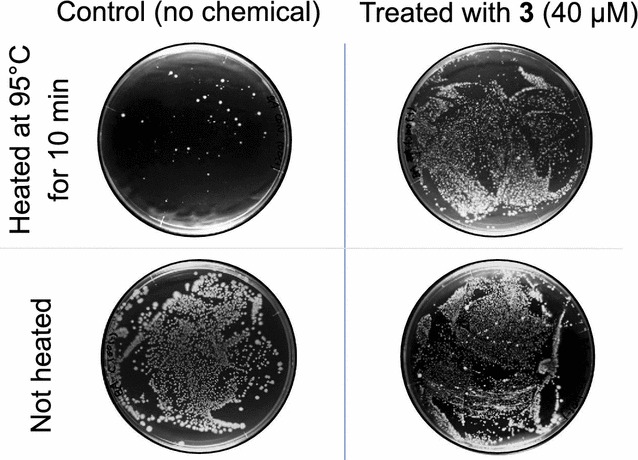
Heat-tolerant assay for sporulated *B. amyloliquefaciens* AHU 2170 with diacetone acrylamide. A 50 µL portion of bacterial cell suspension exposed to 40 µM diacetone acrylamide, incubated for 48-h, and 100-times diluted with Milli-Q water, was kept at 95 °C for 10 min and spread over a 9-cm nutrient broth agar plate. Incubation at 30 °C for 48 h. Two “control” plates (*left*) are without any chemical treatments, while two plates of “not heated” (*bottom*) are without heating treatments

As compound **1** with the free amine is relatively unstable, two amide derivatives *N*-acetyldiacetonamine (**2**) and diacetone acrylamide (**3**) more stable than **1** were also tested for sporulation induction assay. Toward *B. amyloliquefaciens* incubated for 48 h, 400 µM of **2** and **3** showed 54 and 69% sporulation frequencies respectively, which were almost equivalent to or obviously higher than that of **1b**. Interestingly, 40 µM of **3** showed 99% sporulation without cell death (Fig. 3). Therefore, the spores obtained by the treatment with 40 µM of **3** were subjected to heat-tolerant assay, and almost all of the spores survived at 95 °C (Fig. 4). Thus, appearance of the *B. amyloliquefaciens* colonies showed that the spores thus induced by **3** and **1c** are genuine endospores resistant to highly adverse conditions.

**Fig. 5:**
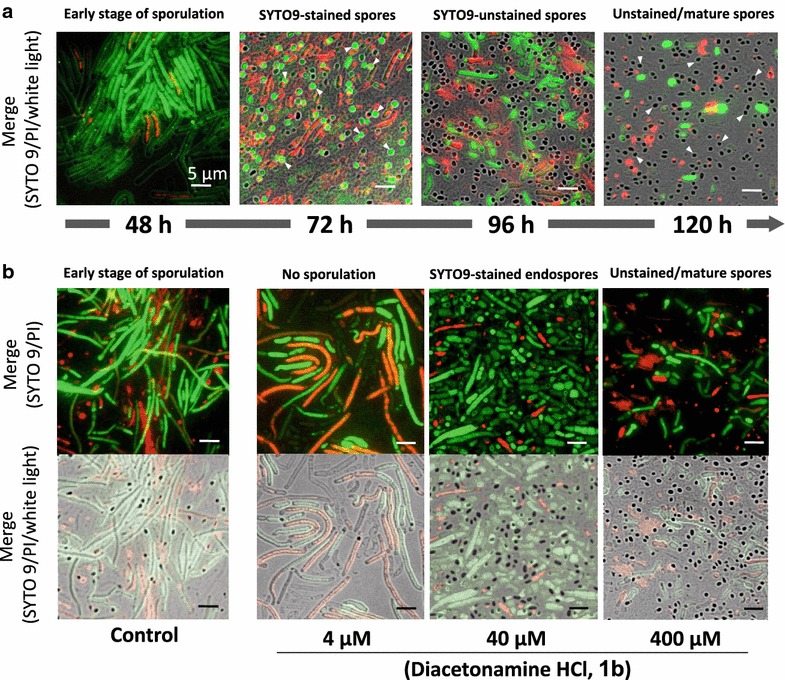
Spore-inducing activity of diacetonamine hydrochloride toward *B. megaterium.* **a** Time-course (48–120 h) of sporulation in *B. megaterium* NBRC 15308 exposed to 400 µM of diacetonamine HCl (**1b**).* Open arrowheads* in photo-images at 72 and 120 h show immature (stained with SYTO 9) and mature (unstained with SYTO 9) endospores, respectively. **b** Dose-dependent manner of **1b** (4, 40, and 400 µM) in the sporulation of *B. megaterium*. At 96-h incubation, microscopic photo-images were recorded. Scale bar = 5 μm

In the vegetative cells of *B. megaterium* treated with 400 µM of **1b**, sporulation induction (36%) was observed at 72-h incubation, in which spores were uniquely stained with SYTO 9 as green fluorescence (arrowheads in 72 h, Fig. 5a). After the incubation for 96–120 h, the spores of *B. megaterium* have changed to mature one unstainable with SYTO 9 (arrowheads in 120 h, Fig. 5a). Thus, relatively slow response of *B. megaterium* to **1b** needed 72-h or longer incubation for the sporulation induction. In addition, dose-dependent manner of **1b** revealed that 40 µM of **1b** induced relatively early stages of sporulation toward vegetative cells of *B. megaterium* after 96-h incubation (55%), while 400 µM of **1b** induced 70% sporulation to *B. megaterium* under the same incubation conditions. Conversely, **1b** at 4 µM did not induce any sporulation at 96 h or even after the incubation for 120 h (Fig. 5b).

**Fig. 6:**
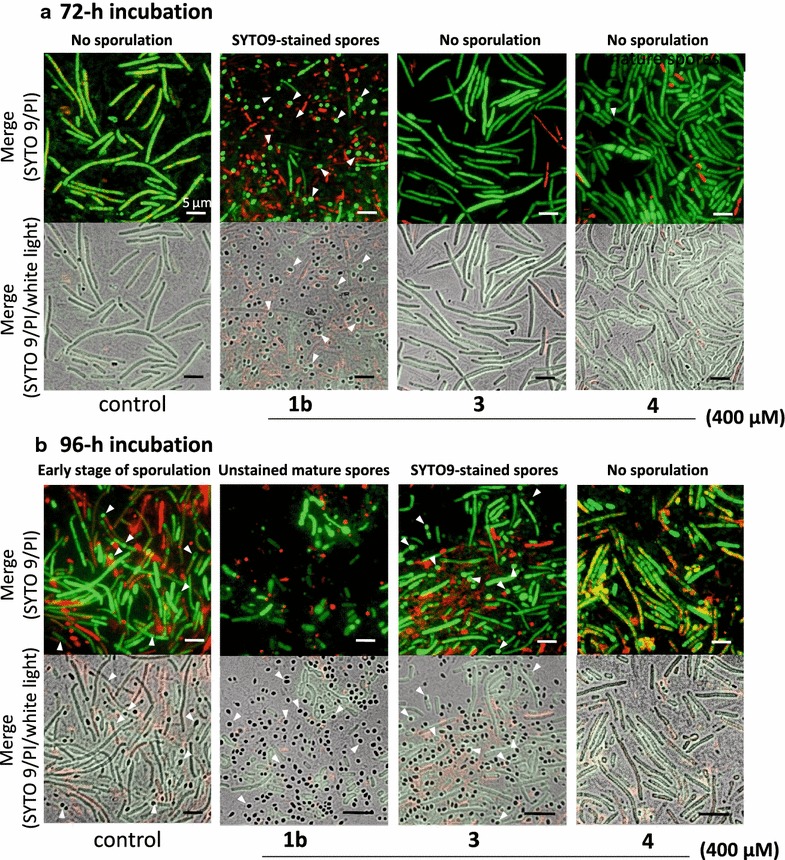
Comparison of sporulation-inducing activities of diacetonamine hydrochloride **(1b)** and diacetone acrylamide **(3)** toward *B. megaterium.* For the sporulation induction assay using *B. megaterium* NBRC 15308, SIF-like activities of the test compounds (**1b**, **3**, and **4**) were examined at 400 µM. **a** Sporulation induction to *B. megaterium* vegetative cells at 72-h incubation. **b** Sporulation induction at 96-h incubation.* Arrowheads* show typical spores abundant in each bacterial cell culture. Scale bar = 5 μm

Diacetone acrylamide (**3**) was also examined its sporulation inducing activity toward *B. megaterium*. Compound **3** at 400 µM did not induce any sporulation at 72-h incubation (Fig. 6a). By the incubation for additional 24 h, **3** led to 66% sporulation frequency that was almost equivalent to **1b** at the same concentration (400 µM) (Fig. 6b). Thus, **3** showed slower sporulation induction than **1b**, leading to almost a 24-h delayed response of *B. megaterium* in the morpho-differentiation. The negative control **4** at the same concentration did not induce any sporulation even at 120 h incubation.

## Discussion

Our study revealed that diacetonamine (**1**) was the main active component in soybean curd residue. Isolation and identification of **1** as SIF were successfully achieved due to our simple and quick bioassay system using the *B. amyloliquefaciens* wild-type strain. In this study, diacetonamine hydrochloride (**1** **b**) showed greater than 34 and 51% sporulation frequencies toward vegetative cells of *B. amyloliquefaciens* at 40 and 400 µM respectively, while 40 µM of diacetone acrylamide (**3**) induced nearly 100% sporulation frequency in the same sporulation assay. Conversely, vegetative cells of *B. megaterium* upon exposure to 400 µM of **1b** needed more than 72-h incubation for sporulation induction. As *B. megaterium* is a *Bacillus* species difficult to induce sporulation (Larsen et al. 2014), the sporulation-inducing activities of **1b** and **3** toward this bacterium are a reliable evidence for **1** as the SIF from soybean curd residue. In addition, the sporulation-inducing activities of **1** and its related compounds toward *B. megaterium* suggested that not only *B. amyloliquefaciens* but several *Bacillus* species are responsive to **1** for sporulation induction.

So far as our literature investigation, an aqueous extract of *Pueraria* radix contained diacetonamine-reineckate (Nakamoto et al. 1975). However, the authors have also shown that **1** in the extract was an artifact yielded by Michael addition of ammonia to mesityl oxide that had been formed by dimerization of acetone used for the extraction solvent (Nakamoto et al. 1976). In contrast, it is most likely that the substance **1** isolated from soybean curd residue is not the artifact spontaneously produced during the isolation process of SIF, simply because we used neither acetone nor ammonia for the extraction and following purification process to obtain Fr-DS-3 (Scheme 1). Indeed, Fr-DS-3 allowed detection of **1** as a unique purple spot on TLC by spraying with *p*-anisaldehyde-sulfuric acid reagent followed by heating. Conversely, there is no evidence whether the compound **1** is a spontaneous artifact during food processing for production of soybean curd residue or a secondary metabolite contained in soybean endosperm. Hence, we never eliminate possibility that **1** is produced as an artifact during boiling and pasting processes of imbibed soybean.

As a report on biological activity of **1** or its derivatives, **1** oxalate-copper has been characterized as an antifungal substance (Ji et al. 2009). Conversely, diacetone acrylamide (**3)** that has been produced on an industrial scale polymerizes with a wide variety of vinyl compounds for a crosslinking agent was characterized as a stable sporulation inducer. Since **3** is water-soluble and more stable than **1**, amide derivatives including **3** would be a promising target for a photoaffinity probe design to detect sporulation signal-sensing proteins linked to the sporulation (Asayama and Kobayashi 1993; Lecca et al. 2016). Because it is known that sporulation event is often linked to the high productivity of antibiotics (Stein 2005), our discovery of diacetonamine (**1**) as a SIF-like compound may contribute to rapid preparation of probiotic materials and exploiting an effective fermentation technology for antibiotic production by *Bacillus* species (Lecca et al. 2016). Further studies on these low molecular compounds towards some other spore-forming *Firmicutes* bacteria will be reported elsewhere.

## Additional file

**Additional file 1: Figure S1.** Activity of methanolic extract from soybean curd residues in antibiotic production.

## Abbreviations

AcOH: acetic acid
DMSO: dimethyl sulfoxide
EI-MS: electron ionization mass spectrometry
EtOAc: ethyl acetate
FD-HR-MS: field desorption high-resolution mass spectrometry
FD-MS: field desorption mass spectrometry
MeOH: methanol
MSG: glutamine-containing minimal salt
NB: nutrient-broth
*n*-BuOH: normal butanol
NMR: nuclear magnetic resonance
SIF: sporulation-inducing factor
TLC: thin-layer chromatography
