# Supplementary material for: Identification of diacetonamine from soybean curd residue as a sporulation-inducing factor toward Bacillus spp
Source: AMB Express. 2017 May 23;7:101. doi: 10.1186/s13568-017-0395-0 (PMC5442031; doi:10.1186/s13568-017-0395-0)
Supplement: Supplementary file 1 — Additional file 1: Figure S1. Activity of methanolic extract from soybean curd residues in antibiotic production. [file 13568_2017_395_MOESM1_ESM.pptx]

## Slide 1
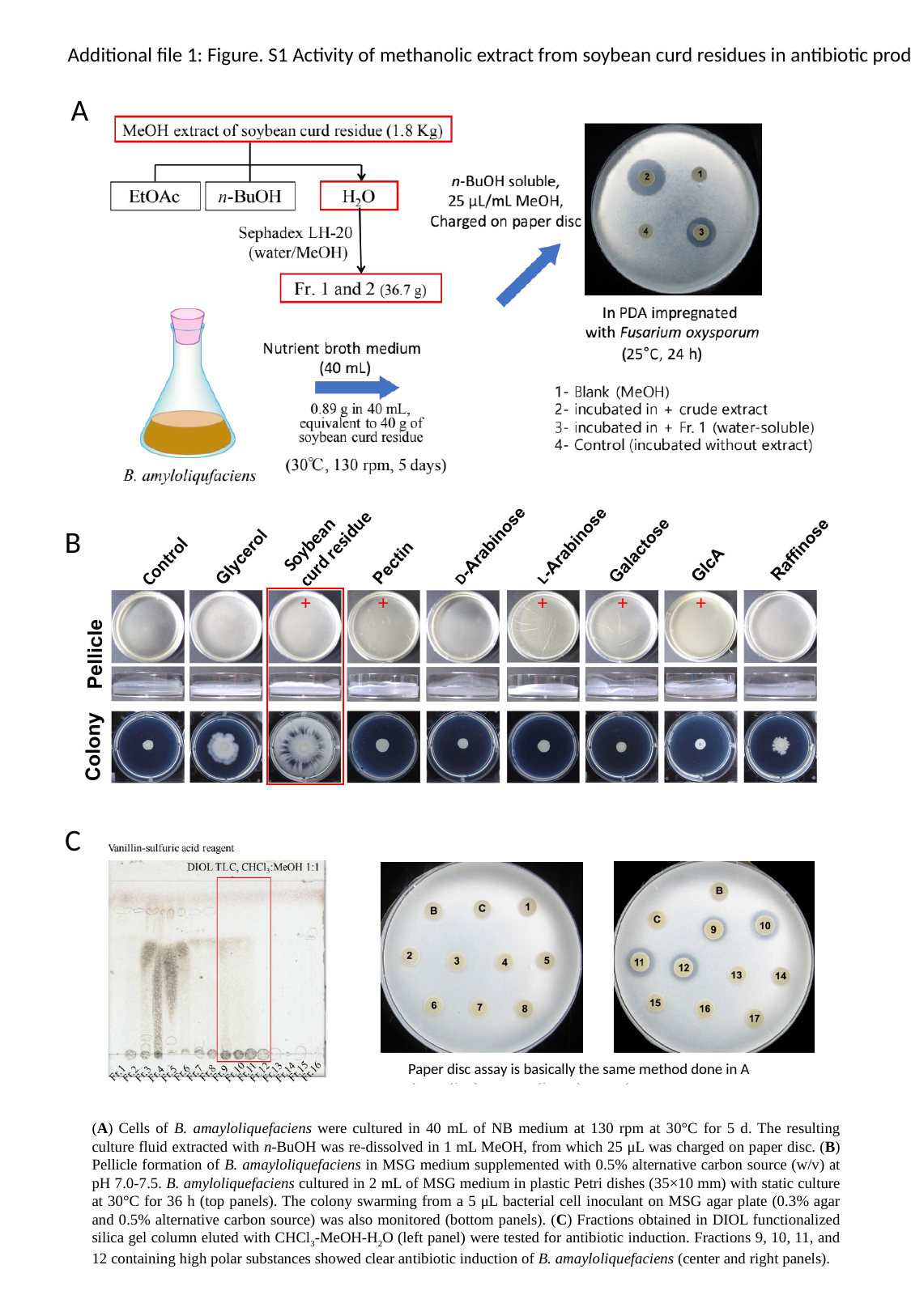

Additional file 1: Figure. S1 Activity of methanolic extract from soybean curd residues in antibiotic production
A
Soybean
curd residue
B
Control
+
+
+
+
+
C
 Paper disc assay is basically the same method done in A
(A) Cells of B. amayloliquefaciens were cultured in 40 mL of NB medium at 130 rpm at 30°C for 5 d. The resulting culture fluid extracted with n-BuOH was re-dissolved in 1 mL MeOH, from which 25 μL was charged on paper disc. (B) Pellicle formation of B. amayloliquefaciens in MSG medium supplemented with 0.5% alternative carbon source (w/v) at pH 7.0-7.5. B. amyloliquefaciens cultured in 2 mL of MSG medium in plastic Petri dishes (35×10 mm) with static culture at 30°C for 36 h (top panels). The colony swarming from a 5 μL bacterial cell inoculant on MSG agar plate (0.3% agar and 0.5% alternative carbon source) was also monitored (bottom panels). (C) Fractions obtained in DIOL functionalized silica gel column eluted with CHCl3-MeOH-H2O (left panel) were tested for antibiotic induction. Fractions 9, 10, 11, and 12 containing high polar substances showed clear antibiotic induction of B. amayloliquefaciens (center and right panels).
